# Osteoarthritis management: Does the pharmacist play a role in bridging the gap between what patients actually know and what they ought to know? Insights from a national online survey

**DOI:** 10.1111/hex.13429

**Published:** 2022-01-08

**Authors:** Andrew J. McLachlan, Peter R. Carroll, David J. Hunter, Tom A. N. Wakefield, Rodney Stosic

**Affiliations:** ^1^ Sydney Pharmacy School, Faculty of Medicine and Health The University of Sydney Camperdown New South Wales Australia; ^2^ Department of Rheumatology, Royal North Shore Hospital The University of Sydney St. Leonards New South Wales Australia; ^3^ Department of Clinical Pharmacology Royal North Shore Hospital St. Leonards New South Wales Australia; ^4^ FiftyFive5 Ultimo New South Wales Australia; ^5^ Bayer Australia Pty., Ltd. Pymble New South Wales Australia

**Keywords:** analgesics, Australia, community pharmacy, health literacy, osteoarthritis, recommendations, screening

## Abstract

**Background:**

Guidelines encourage engagement in self‐care activities for osteoarthritis (OA), but there are gaps in consumers' knowledge about suitable choices for self‐care. Community pharmacists are in an ideal position to contribute to OA management through screening and supporting evidence‐based pain management choices. Prior research established an association between health literacy and advice‐seeking and appropriateness of analgesics choices (both lower in participants with limited health literacy) amongst people living with OA. This article explores the implications of these data for pharmacists in OA management.

**Methods:**

A national online survey was conducted amongst 628 adults aged 45–74 years, currently residing in Australia, with self‐reported symptoms of OA. All data were collected using a customized online questionnaire, which was completed only once. ‘Self‐reported symptoms of OA’ was based on six validated screening questions to identify people with OA without a formal clinical diagnosis.

**Results:**

Respondents matched the typical profile of people diagnosed with OA; more than half were female (56%), knees (59%) and hips (31%) were the primary affected joints and 74% were either overweight or obese. Self‐identification of OA was limited (41%). Overall, 38% self‐managed their pain, and limited health literacy was associated with less advice‐seeking. Efficacy and ease of use were the main reasons cited for prompting use across all classes of nonprescription analgesic, with less than 20% reporting recommendation from a pharmacist. Participants were managing their pain with an average of 1.74 (95% confidence interval: 1.60–1.88) analgesics, but 73% reported inadequate pain relief and 54% had disrupted sleep.

**Conclusion:**

Our findings highlight three key themes: lack of self‐identification of OA, suboptimal pain relief and limited use of the community pharmacist as a source of management advice. Equipping community pharmacists with tools to identify OA could bridge this gap. More research is needed to determine if it will improve consumers' ability to appropriately manage OA pain.

**Patient or Public Contribution:**

Consumers living with OA contributed to the study outcomes, reviewed the survey questionnaire for face validity and advised on plain language terminology.

## 1 INTRODUCTION

Osteoarthritis (OA), a complex, chronic health problem, is associated with significant burden.^1^ Australian National Health Survey data collected in 2017–2018 estimated that one‐fifth of Australians over the age of 45 years had OA.^2^ Global data predict OA to become one of the most prevalent diseases in populations from high‐income countries,^1^ with the suggestion that by 2032, almost 30% of the population aged 45 years are expected to have doctor‐diagnosed OA.^3^

Current OA management focuses on symptomatic relief and reducing disability progression, via a combination of lifestyle, nonpharmacological, pharmacological and surgical approaches tailored to the needs of the individual.^4^, ^5^, ^6^ To further facilitate lifestyle and nonpharmacological approaches, established guidelines encourage engagement in self‐care activities.^4^, ^7^ Proposed strategies to mitigate the future burden of OA suggest that there is a need for ‘better awareness, especially of the risk factors, and early diagnosis and treatment of OA together with the improvement of healthcare infrastructure for managing the increasing number of patients with OA’.^1^

Health literacy has been highlighted as an important component of patient engagement self‐management strategies.^8^, ^9^, ^10^, ^11^ There are negative relationships between health literacy and diagnosis comprehension,^12^, ^13^ health outcomes^14^ and use of healthcare services,^15^ and limited health literacy is identified as an obstacle to accessing primary care services.^16^

Adequate health literacy has previously been shown to be integral to patients' decisions to adopt and engage with OA self‐management strategies.^11^ However, data exploring this issue from the perspective of the patient are limited.^11^, ^17^ A recent survey, conducted amongst adults with self‐reported symptoms of OA, investigated the participants' perceptions of pain and their use of self‐management strategies for pain relief.^18^ The study reported a significant association between health literacy and the appropriate selection of recommended self‐management strategies and highlighted participants suboptimal knowledge about suitable pain relief choices. Recognizing that the pharmacist is well placed to provide counselling to help improve patients' knowledge about and correct use of medicines,^19^, ^20^ this paper explores this study, focusing on the findings of relevance to the community pharmacist.

## 2 METHODS

The methods have been published in detail elsewhere.^18^ Briefly, a national online survey was conducted amongst adults aged 45–74 years, currently residing in Australia, with self‐reported symptoms of OA. The cohort was derived from an Australian accredited research‐only online panel (Dynata). Participants were incentivized through the receipt of panel points and provided informed consent before commencing the survey. All data were self‐reported and collected using a customized online deidentified questionnaire, which was completed only once. Ethical clearance was received (Bellberry Human Research Ethics Committee; Eastwood, SA, Australia: HREC2020‐05‐494‐A1).

The key study eligibility criterion ‘self‐reported symptoms of OA’ was based on six screening questions designed specifically to capture a representative population of people with OA without seeking formal evidence of a clinical diagnosis. Three of the screening questions (1) ‘age 45 years or over’, (2) ‘has activity‐related joint pain’ and (3) ‘has either no morning joint‐related stiffness or morning stiffness that lasts no longer than 30 min’ were based on the definition of clinically diagnosed OA supported by the National Institute for Health and Clinical Excellence Guidelines for OA^6^ and the Royal Australian College of General Practitioners guideline for the management of hip and knee OA.^4^ Three additional screening questions related to (4) ‘no trauma in the previous 3 months’, (5) ‘a minimum duration of pain (>3 months)’ and (6) ‘a minimum pain intensity (>3/10 on an 11‐point visual analogue scale [VAS])’^21^ ‘further defined current OA and helped to rule out other causes.’^22^

The remainder of the questionnaire comprised validated tools to evaluate health literacy,^23^, ^24^, ^25^ health‐related quality of life,^26^, ^27^ OA symptoms, impact and comorbidities,^28^, ^29^, ^30^, ^31^, ^32^, ^33^, ^34^, ^35^, ^36^ medication beliefs and adherence^37^, ^38^, ^39^ and other questions developed empirically through review of the literature.^22^, ^40^, ^41^, ^42^ A group of consumers living with OA contributed to selecting the study outcomes, reviewed the survey questionnaire for face validity and provided advice on plain language terminology. Literature‐based quality indicators^4^, ^5^, ^43^ were predefined in the protocol based on a traffic light system (green, amber, red) and used to categorize the appropriateness of the respondents' choices for OA self‐care. Within this system, *green* represented core strategies and/or those with a strong recommendation or conditional recommendation (Levels 1A, 1B, 2), *amber* strategies with a conditional recommendation neutral or against (Levels 3, and 4B) and *red* strategies, which are not recommended (Levels 4A and 5).

The primary analysis population comprised all participants who fulfilled the criteria for self‐reported symptoms of OA. Four prespecified, but not mutually exclusive, subpopulations were defined to account for differences in management recommendations amongst participants with comorbidities: OA and no comorbidity, OA plus gastrointestinal comorbidities, OA plus cardiovascular comorbidities and OA plus widespread pain and/or depression.

The average proportion of green, amber and red management strategies that a respondent was using provided a numeric measure of the appropriateness of their self‐care choices, and health literacy was measured using a categorical variable that classified each person's health literacy into low (score ≤ 32), moderate (score: 33–38) or high (score > 38) using validated cut‐off criteria.^25^, ^44^ For each participant, the number of appropriate strategies (based on the traffic light system, green/amber/red) they reported using were first summed and then divided by the total number of strategies recommended for their level of comorbidity. A score of 0 was used to indicate that they are not using any green/amber/red strategies, a score of 0.5 was used to indicate that they are using half of the green/amber/red strategies and a score of 1 was used to indicate that they were using all green/amber/red strategies. A higher score was, therefore, indicative of more appropriate choices for green strategies, but less appropriate choices for red strategies. A one‐way multivariate analysis of variance test, with the three management appropriateness metrics as the dependent variables and health literacy as the independent variable, was used to test for statistical significance.

Baseline variables and outcome measures were analysed among the primary and subpopulations. Continuous variables were summarized descriptively, and differences were analysed using two‐sample *t*‐tests and categorical variables were summarized as contingency tables using sample sizes and percentages with two‐sided 95% confidence intervals (CIs) and analysed using two‐sample *z*‐tests. A *p*‐value less than .05 was considered significant. All statistical analyses were generated using Q‐research software (Ver 5.9.7.0; Displayr).

## 3 RESULTS

Out of the total pool of respondents (*N* = 6800), 6348 were in the target age range (45–74 years) and answered all screening questions. Of these, 697 (10%) fulfilled all of the screening criteria for self‐reported OA, and 69 withdrew consent, leaving a population of 628 eligible participants (Table 1). Detailed demographics for the primary and secondary populations are provided in Table S1.

**Table 1:** Participant demographics

	Primary	HLS‐12: Categories^a^		
	OA (all)	Low	Moderate	Excellent
	*N* = 628	*N* = 100	*N* = 317	*N* = 211
Sex				
Male	43.6%	56.0%	44.8%	36.0%
Female	56.4%	44.0%	55.2%	64.0%
Age (years)				
45–49	11.9%	13.0%	12.0%	11.4%
50–54	15.3%	15.0%	14.2%	17.1%
55–59	14.0%	18.0%	13.6%	12.8%
60–64	21.3%	20.0%	21.8%	21.3%
65–69	20.7%	18.0%	21.8%	20.4%
70–74	16.7%	16.0%	16.7%	17.1%
Geographic location				
Major cities of Australia	73.6%	79.0%	71.6%	73.9%
Inner regional Australia	19.3%	15.0%	21.1%	18.5%
Remote/outer Regional Australia	7.2%	6.0%	7.2%	7.6%
Ethnicity				
White/Caucasian	92.5%	92.0%	91.2%	94.8%
Asian	3.3%	4.0%	4.1%	1.9%
Middle Eastern	1.1%	1.0%	0.9%	1.4%
Chinese	0.3%	0.0%	0.6%	0.0%
Other	2.7%	3.0%	3.2%	1.9%
Country of birth				
Australia	75.3%	71.0%	75.1%	77.7%
Other	23.7%	29.0%	23.3%	21.8%
Rather not say	1.0%	0.0%	1.6%	0.5%
Languages spoken at home other than English				
No, English only	94.3%	90.0%	94.3%	96.2%
Yes, other	5.7%	10.0%	5.7%	3.8%
Marital status				
Never married	13.7%	16.0%	13.2%	13.3%
Married/living with partner	63.2%	66.0%	61.5%	64.5%
Widowed/divorced/separated	23.1%	18.0%	25.2%	22.3%
Education				
Less than Year 12	18.9%	21.0%	20.5%	15.6%
Senior secondary school certificate of education	18.3%	12.0%	21.5%	16.6%
Vocational qualification	33.6%	33.0%	31.2%	37.4%
University degree or higher	28.2%	34.0%	25.6%	29.4%
Rather not say	1.0%	0.0%	1.3%	0.9%
Household income (prior year, pretax)				
Under $10,000	1.4%	0.0%	1.6%	1.9%
$10,000–$49,999	34.7%	27.0%	37.9%	33.6%
$50,000–$99,999	32.0%	35.0%	30.0%	33.7%
$100,000 or more	21.3%	28.0%	21.5%	18.0%
Rather not say	10.5%	10.0%	9.1%	12.8%
Employment status				
Employed (full time or part time)	37.9%	44.0%	37.9%	35.1%
Unpaid work (volunteering)	2.7%	2.0%	3.2%	2.4%
Caregiver (children, elderly)	4.1%	2.0%	4.4%	4.7%
Student (full time or part time)	0.8%	2.0%	0.3%	0.9%
Retired	44.1%	39.0%	43.5%	47.4%
Other	10.4%	11.0%	10.7%	9.5%
Medical insurance				
No	42.2%	47.0%	41.6%	40.8%
Yes	57.8%	53.0%	58.4%	59.2%

Abbreviations: HL, health literacy; HLS‐12, European Health Literacy Survey Questionnaire: Short‐Form; OA, osteoarthritis.

^a^ HLS‐12 score categories: Low (minimal/inadequate, less than 32), moderate (33–38) and excellent (more than 38). Statistically significant (*p* < .05) differences: Red indicates a significantly lower average versus the other health literacy categories, and blue indicates a significantly higher average versus the other health literacy categories.

### 3.1 Health literacy and appropriateness of management choices

The proportions of participants classified as having low, moderate or high health literacy were similar in the overall population and the secondary analysis populations. A significantly higher proportion of females were categorized as having high health literacy than were males (Table 1). The primary endpoint analysis showed evidence of a statistically significant interaction between health literacy and overall appropriateness of management strategies (*p* < .001).^18^

### 3.2 Baseline characteristics, pain symptoms and self‐recognition of OA

The majority of participants (82.5%) were nonsmokers; almost three‐quarters were overweight or obese and health‐related quality of life was generally good (Table 2). Participants reported having current pain in 4.1 joints; the most frequent location of joint pain was in the lower body, and the majority (82.1%) had been experiencing joint pain for more than a year (Table 2).

**Table 2:** Participants' baseline characteristics

	Primary population (*N* = 628)
Smoking status	
No, never	45.7%
Yes, quit more than a year ago	35.4%
Yes, quit in the last 12 months	1.4%
Yes (social)	4.0%
Yes (current)	13.5%
Body mass index (BMI)	
Mean BMI (kg/m^2^)	29.5
Underweight (below 18.5)	1.3%
Normal (18.5–24.9)	23.9%
Overweight (25–29.9)	34.4%
Obese (30 and above)	40.4%
Health‐related quality of life (PROMIS‐10)	
General health	
Poor/fair	24.2%
Good	43.5%
Very good/excellent	34.15%
Physical health	
Poor/fair	27.9%
Good	35.0%
Very good/excellent	37.1%
Mental health	
Poor/fair	31.7%
Good	22.9%
Very good/excellent	45.4%
Self‐reported joint pain	
Previous joint replacement surgery	11.0%
Mean number of joints currently affected by pain	4.1
Location of joint pain	
Neck	18.5%
Shoulders	15.4%
Elbows	9.4%
Hips	31.1%
Hands	27.5%
Knees	59.1%
Feet	26.4%
Duration of joint pain	
3–6 months	6.2%
6–12 months	11.6%
1–5 years	35.8%
5–10 years	20.5%
More than 10 years	25.8%

**Figure 1:**
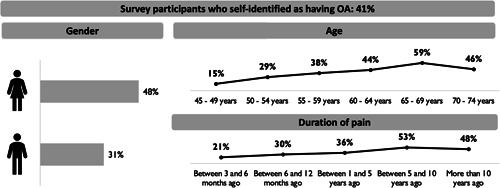
Self‐identification of osteoarthritis (OA) amongst the primary analysis population

All participants fulfilled the study eligibility criterion for ‘self‐reported symptoms of OA’, but when presented with a list and asked to indicate if they had any current health conditions or were taking medications/receiving treatment for current conditions, 40.8% self‐identified OA and, amongst those, only half (52.0%) reported currently taking medication and/or receiving treatment for OA. Women, participants aged 55–74 years and those who had experienced pain for longer (5+ years) were more likely to self‐identify as having OA (Figure 1).

Participants reported an average pain intensity of 5.9 (95% CI: 5.8–6.0) at its worst and 4.7 (95% CI: 4.6–4.9) in the past 7 days, on a scale of 0–10. The corresponding data from 0 to 100 VAS scales used in validated clinical tools for hip (HOOS‐12)^28^, ^29^ and knee (KOOS‐12)^28^, ^30^ OA were similar: average hip pain intensity (64.0; 95% CI: 62.1–65.9) and average knee pain intensity (63.5; 95% CI: 61.9–65.1). Participants were managing their pain with an average of 1.44 (95% CI: 1.32–1.57) self‐care activities, 1.74 (95% CI: 1.60–1.88) analgesics and 1.01 (95% CI: 0.90–1.12) complementary medicines, but the majority (72.9%) reported inadequate pain relief (VAS score ≥ 4) in the past week and over half (54.1%) had disrupted sleep, with 10.7% reporting disrupted sleep on 3 or more nights each week in the past month.

### 3.3 Resources used for pain management

The general practitioner/family doctor was the main healthcare provider from whom advice about current pain management was received (Table 3). Almost 4 out of 10 (37.9%) participants reported that they were not receiving help from anyone to manage their pain (Table 3), and a similarly high proportion (44.9%) reported that they used the internet search as a source for advice or information about how to manage their joint pain. There was an overall association between health literacy and advice seeking, but the differences were not statistically significant. When compared to participants classified as having moderate (64%) and high (63%) health literacy, fewer (54%) participants with low health literacy reported having sought any advice for pain management (Table 3).

**Table 3:** Resource utilisation

	Primary	HLS‐12: Categories^a^		
	OA (all)	Low	Moderate	Excellent
	*N* = 628	*N* = 100	*N* = 317	*N* = 211
Resource utilisation (who is currently helping you to manage this pain?)				
General practitioner	49.2%	43.0%	50.8%	49.8%
No‐one	37.9%	46.0%	35.6%	37.4%
Allied healthcare provider^b^	21.9%	21.0%	22.4%	21.8%
Pain specialist/orthopaedic surgeon	11.8%	9.0%	12.0%	12.8%
Pharmacist	3.5%	4.0%	4.7%	1.4%
Friend/family member	2.4%	3.0%	1.6%	3.3%
Other	2.1%	2.0%	1.9%	2.4%

Abbreviations: HL, health literacy; HLS‐12, European Health Literacy Survey Questionnaire: Short‐Form; OA, osteoarthritis.

^a^ HLS‐12 score categories: Low (minimal/inadequate, less than 32), moderate (33–38), excellent (more than 38) statistically significant (*p* < .05) differences: Red indicates a significantly lower average versus the other health literacy categories and blue indicates a significantly higher average versus the other health literacy categories.

^b^ Allied health care provider = physiotherapist, chiropractor, osteopath, massage therapist.

### 3.4 Awareness, choice and use of nonprescription analgesics

There were high levels of awareness of different classes of analgesics that could be purchased without a prescription, and paracetamol was reported to be the most frequently used of these analgesics (Table 4). The two main sources of recommendation for use of nonprescription analgesics were the family doctor and the pharmacist, and these healthcare professionals prompted current use in around half of the respondents. Around 30% of participants reported that no one had recommended the use of their current analgesic; efficacy and ease of use were the main reasons cited for prompting use across all classes of non‐prescription analgesics (Table 4). Rates of adherence were suboptimal; less than two in five participants were found to have a high level of adherence irrespective of the nonprescription analgesic being used. Around three‐quarters of participants reported being satisfied, or highly satisfied, with the pain relief achieved from their analgesic (Table 4). The pharmacy was the primary place to purchase nonprescription analgesics, accounting for 75%–80% of purchases depending on the analgesic, with 25%–35% of purchases being made in the supermarket; some people reported purchasing from both places.

**Table 4:** Awareness, choice and use of nonprescription analgesics

	Paracetamol	Oral NSAID	Paracetamol/ibuprofen combination	Topical NSAID	Other topical^a^
	*N* = 297	*N* = 120	*N* = 41	*N* = 85	*N* = 100
Awareness and use					
Currently using	47.3%	19.1%	6.5%	13.5%	15.9%
Used within the last year	25.2%	23.1%	13.4%	20.1%	18.5%
Used more than a year ago	10.8%	18.0%	9.4%	16.4%	14.5%
Aware of, but not tried	14.5%	31.1%	44.1%	34.1%	33.1%
Reasons prompting use					
The doctor prescribed it	33.7%	23.3%	19.5%	21.2%	22.0%
A pharmacist recommended it	18.9%	18.3%	26.8%	32.9%	24.0%
Effective pain relief	41.8%	52.5%	46.3%	41.2%	40.0%
Easy to use	39.1%	34.2%	41.5%	38.8%	42.0%
Convenience	24.2%	21.7%	26.8%	18.8%	23.0%
Sources of recommendation					
General practitioner	57.9%	43.3%	43.9%	43.5%	42.0%
No one	28.3%	33.3%	24.4%	27.1%	23.0%
Pharmacist	12.5%	15.8%	17.1%	12.9%	16.0%
Pain specialist/orthopaedic surgeon	8.1%	7.5%	12.2%	4.7%	2.0%
Friend/family member	8.8%	11.7%	14.6%	15.3%	19.0%
Allied healthcare provider^b^	6.1%	2.5%	12.2%	10.6%	12.0%
Other	1.0%	2.5%	4.9%	4.7%	1.0%
Adherence^c^					
Low	19.5%	20.8%	24.4%	27.1%	24.0%
Medium	59.6%	56.7%	61.0%	55.3%	57.0%
High	20.9%	22.5%	14.6%	17.6%	19.0%
Satisfaction^d^					
Low	29.3%	21.7%	12.2%	24.7%	25.0%
Medium	44.8%	50.0%	43.9%	50.6%	44.0%
High	25.9%	28.3%	43.9%	24.7%	31.0%
Purchased from where?					
Pharmacy/online pharmacy	77.8%	76.7%	73.2%	82.4%	84.0%
Supermarket	34.3%	35.8%	29.3%	30.6%	25.0%

Abbreviation: NSAID, nonsteroidal anti‐inflammatory drug.

^a^ Topical pain relief preparations include arnica, camphor, menthol, methyl salicylate, capsaicin.

^b^ Allied health care provider = physiotherapist, chiropractor, osteopath, massage therapist

^c^ Adherence was assessed using the Morisky Medication‐Taking Adherence Scale; adherence was calculated as follows, score of 4 = high, 3 or 2 = intermediate, and 1 or 0 = low.

^d^ Satisfaction was assessed using a categorical scale and grouped for presentation; high = extremely/very satisfied, intermediate = satisfied, low = somewhat satisfied /not at all satisfied.

## 4 DISCUSSION

A recent online survey amongst adults with self‐reported symptoms of OA has identified limited self‐recognition of OA, suboptimal knowledge about suitable choices for OA self‐care and a statistically significant interaction between health literacy and overall appropriateness of management strategies.^18^ Further exploration of this data has identified a high level of self‐reliance for pain management advice—38% of participants were not receiving help from anyone and 45% were using the internet as a source of advice—an association between not seeking advice and inadequate health literacy, and ease of use as the key driver prompting the trial of a nonprescription analgesic.

The study results are strengthened by the use of a wide selection of validated assessment tools and the large prospective sample of more than 600 respondents but limited by the potential for bias from the online survey methodology.^18^ With approximately 19% of the population anticipated to fulfil the eligibility criteria, it was expected that 3156 respondents would be required to ensure a minimum sample size of 600 participants for statistical analysis. A number of patients (*n* = 2987) failed study entry because they had reported an injury within the last 3 months. Excluding these respondents, 21% of the sample would have qualified for the survey, closely matching the original sample estimates and supporting its representativeness. Participant characteristics were consistent with the profile of patients with OA,^3^ including gender, age distribution, the location of the joints affected and the presence of comorbidities. Furthermore, demographic data, including ethnicity, geographical location, marital status, education, income and medical insurance, demonstrated comparable representativeness to the Australian population.

Others have suggested that patients with early knee OA may lack sufficient knowledge to recognize their condition.^45^ Our results corroborate this, and suggest that a lack of self‐recognition of OA may also lead to poor self‐management. Despite meeting guideline‐endorsed clinical criteria for a diagnosis of OA, three out of every five participants did not self‐identify that they had OA and the majority (72%) reported inadequate pain relief in the past week. Amongst the nonprescription analgesics currently being used, paracetamol was most widely used (47% of participants), but 3 in every 10 participants reported low satisfaction with the outcomes. While historically paracetamol has been regarded as a first‐line analgesic for the management of OA pain, there is high‐quality evidence that it provides only modest pain relief for people with knee or hip OA (mean pain relief on a 0–10 scale of 0.3 points; 95% CI: –0.6 to –0.1 point decrease in the pain score).^46^ This is below the minimum clinically important difference, which is typically defined as 10% or a 1‐point change on a 0–10 point scale.^47^ Current guidelines for the management of hip and knee OA provide a neutral recommendation for paracetamol and topical nonsteroidal anti‐inflammatory drugs (NSAIDs) and a conditional recommendation for the use of oral NSAIDs.^4^, ^48^

Overall, two in every five respondents reported that they were managing their joint pain on their own. Participants reported high levels of awareness of different classes of nonprescription analgesics. This may explain why only 17% had sought advice from the community pharmacist specifically about the use of this class of analgesics to manage their joint pain, and also why features such as efficacy, convenience and ease of use were cited more frequently than was pharmacist recommendation as a reason for having selected a particular analgesic.

The medicines scheduling in Australia are such that some medicines are permitted to be purchased in general sales outlets, while others are restricted to purchase only in pharmacies or after receipt of pharmacist advice. The nonprescription analgesics named in the survey can be purchased in either the pharmacy or the supermarket, but there are pack size restrictions (less than 20 tablets) on the products available in the supermarket. Place of purchase data show that the pharmacy is still the main place for purchase analgesics, but up to 35% of users of nonprescription analgesics reported purchasing from a supermarket. The main reasons for choice of analgesic were efficacy and ease of use, and many respondents did not seek any advice on recommendation. This might imply that a proportion of respondents had been using whatever analgesic they had available rather than seeking out advice for an analgesic specifically for their joint pain. Further research would be needed to better define these parameters and to determine if there is any correlation between analgesic choice based on convenience and suboptimal pain control.

Adequacy of health literacy is associated with a patient's ability to access, understand, appraise and interpret health information.^49^ Patients lacking these core competencies in health literacy are limited in their ability to apply information to their current medical or clinical issues, their risk factors for health and to determinants of health in their social and physical environments. All of these can impact decision‐making in relation to healthcare needs, disease prevention and health promotion. Having adequate health literacy has been highlighted as a key factor in patients' decisions to adopt and engage with OA self‐management strategies.^11^ Health literacy has the potential to influence the appropriateness of self‐care strategies and could therefore impact outcomes achievable via patient‐directed OA management.^8^, ^9^, ^50^ The primary endpoint analysis from the study highlights this to be of particular relevance.^18^ Knowledge about suitable choices of analgesics for joint pain management was suboptimal. Use of green (evidence levels 1A, 1B and 2) analgesics was low across all three health literacy groups, although there was a numerical trend for the use of more green strategies with increasing health literacy classification. However, amongst the red (evidence levels 4A, 5) analgesics, there was also a numerical trend for the use of more of these strategies with increasing health literacy classification, suggesting that higher health literacy does not always translate into appropriate choices. Amongst participants classified as having high health literacy, use of supplements and/or combinations of analgesics for which the evidence base is either insufficient or unsupported (per the quality indicators^4^, ^5^, ^43^ predefined in our protocol) was a key component of these poor choices.

It has previously been suggested that there is a need for interventions that improve patients' knowledge of OA and their ability to self‐manage it, whilst tailoring management to the needs of the individual patient.^42^ However, this only becomes practical after the patient had been diagnosed. Many patients view musculoskeletal pain as a normal part of ageing.^51^ It is deemed to be of less importance within the hierarchy of conditions for which healthcare support is sought,^52^ with lack of natural resolution of pain cited as a reason for seeking initial healthcare input.^53^

Community pharmacists are frontline, accessible healthcare professionals.^54^ It is already established that screening for chronic diseases in a community pharmacy can facilitate earlier diagnosis and identification of risk factors.^55^ Through their traditional role as dispensers of medications and providers of medicines and health advice, community pharmacists have become a trusted source of information and are positioned to provide patients with healthcare support.^56^ In recent years, the role of the community pharmacist has expanded, such that it now incorporates medication assessment and patient education as well as screening and management advice for a number of conditions with demonstrated improvements in patient outcomes.^57^ Prior research has established that, when provided with a simple screening questionnaire, community pharmacists are able to identify more than 80% of patients with knee pain who have undiagnosed knee OA.^58^ Canadian guidelines suggest that the community pharmacist can screen patients aged 45 years or older for symptoms of OA, then triage these customers by providing education, treatment recommendations in accordance with current OA guidelines and referral to other healthcare professionals as appropriate.^54^ In this study, early OA screening was based on guidelines supporting that OA can be diagnosed clinically without the need for diagnostic tests or imaging^6^ through the use of three criteria (age 45 years or older, the presence of activity‐related joint pain and no morning joint‐related stiffness or stiffness that lasts less than 30 min). Our survey utilized these three screening criteria and then expanded this with three additional questions (minimum duration of pain > 3 months, minimum pain intensity > 3/10 and no recent trauma) to better help to establish current OA and rule out other causes, and has demonstrated it to be an effective screening tool for identifying early OA.^22^

However, our research highlights a gap in the use of the community pharmacist as a resource for advice about OA pain and its management. The data demonstrate that overall, only 3.5% of respondents had sought pain management advice from the pharmacist. In addition, there was an association with health literacy, with the data showing that more people with low health literacy reported not seeking pain relief advice from anyone. It is vital that pharmacists recognize the limitations of low health literacy, in particular, how they relate to the lack of willingness of a consumer to proactively seek advice and to be able to act upon that advice. Prior research identified an association between lack of dialogue and health literacy and has called for increased engagement between the pharmacist and the consumer.^59^ Use of a simple screening tool to establish a diagnosis of OA could be used to open up such a dialogue, enabling the pharmacist to educate the customer about the condition, conduct a medication review, provide advice on evidence‐based analgesic choices and refer as required. Whilst this strategy may have the potential to bridge the gap between what patients ought to know and what they actually know, research will be needed to determine its uptake and impact on care outcomes.

## 5 CONCLUSION

Almost three in five patients with diagnosable OA may not be actively seeking medical care. Pharmacists are a trusted source of advice, are in a unique position to be able to appraise the current medications people are taking and understand the potential for drug–drug interactions; yet, there is evidence of only limited involvement in joint pain management in the community pharmacy setting. Equipping community pharmacists with tools to identify OA may help to bridge this gap by creating a dialogue about OA. More research is needed to determine if such a strategy would lead to any improvements in consumers' knowledge or their ability to appropriately manage early OA pain.

## CONFLICT OF INTERESTS

All authors have completed the ICMJE uniform disclosure form at www.icmje.org/coi_disclosure.pdf and declare: David J. Hunter reports personal fees and other support from Bayer Australia Ltd., Consumer Healthcare during the conduct of the study and personal fees from Merck Serono, Novartis, Bayer, Tissuegene and TLCBio outside the submitted work. Andrew J. McLachlan reports personal fees and other support from Bayer Australia Ltd., Consumer Healthcare during the conduct of the study and research funding to the Sydney Pharmacy School from GSK to support a PhD scholar under his supervision, outside the submitted work. Peter R. Carroll reports personal fees and other support from Bayer Australia Ltd., Consumer Healthcare during the conduct of the study. Tom A. N. Wakefieldreports receiving personal fees from his employer (Fifty Five 5 Pty., Ltd.), who were paid consulting fees by Bayer Australia Ltd., Consumer Healthcare during the conduct of the study and nonfinancial support provided by Bayer Australia Ltd., Consumer Healthcare during the preparation of the manuscript. Rodney Stosic is an employee of Bayer Australia Ltd. Andrew J. McLachlan (the manuscript's guarantor) affirms that the manuscript is an honest, accurate and transparent account of the study being reported; that no important aspects of the study have been omitted; and that any discrepancies from the study as planned (and, if relevant, registered) have been explained.

## AUTHOR CONTRIBUTIONS

The following provides a summary of the contributions of each of the authors. David J. Hunter, Andrew J. McLachlan, Peter R. Carroll, Tom A. N. Wakefield and Rodney Stosic conceived the concept of this study and designed the study. David J. Hunter conceived the initial survey questions upon which this study is based; Andrew J. McLachlan, Peter R.Carroll, Tom A. N. Wakefield and Rodney Stosic further refined the survey questions before ethics review and approval. David J. Hunter, Andrew J. McLachlan, Peter R. Carroll and Tom A. N. Wakefield were involved in the conduct of the study; data collection was undertaken by FiftyFive5Pty Ltd. David J. Hunter, Andrew J. McLachlan, Peter R. Carroll and Rodney Stosic guided the analysis of the data and Tom A. N. Wakefield conducted the analysis of the data. David J. Hunter, Andrew J. McLachlan, Peter R. Carroll, Tom A. N. Wakefield and Rodney Stosic contributed to the interpretation of the results. David J. Hunter, Andrew J. McLachlan, Peter R. Carroll, Tom A. N. Wakefield and Rodney Stosic contributed to an outline from which the manuscript was drafted. David J. Hunter, Andrew J. McLachlan, Peter R. Carroll, Tom A. N. Wakefield and Rodney Stosic revised the manuscript critically for important intellectual content and gave final approval of the version to be published. Andrew J. McLachlan accepts full responsibility for this study, had access to the data and controlled the decision to publish.

## Supporting information

Supporting information.

## Data Availability

Extra data are available upon reasonable request and with the permission of Bayer Australia Ltd., Consumer Healthcare by emailing: rodney.stosic@bayer.com. A summary report has been provided to the ethics review committee. There are no plans to disseminate the results to the study participants, except upon request.
