# Supplementary material for: Osteoarthritis management: Does the pharmacist play a role in bridging the gap between what patients actually know and what they ought to know? Insights from a national online survey
Source: Health Expect. 2022 Jan 8;25(3):936–46. doi: 10.1111/hex.13429 (PMC9122430; doi:10.1111/hex.13429)
Supplement: Supplementary file 1 — Supporting information. [file HEX-25--s001.docx]

**Supplementary materials**

Table S1. Participant demographics

|  | **Primary** | **Secondary analysis populations** | | | |
| --- | --- | --- | --- | --- | --- |
|  | **OA (all)** | **OA** | **OA + CV** | **OA + GI** | **OA + Pain/ Depression** |
|  | **N=628** | **N=263** | **N=257** | **N=29*** | **N=196** |
| **Sex** |  |  |  |  |  |
| Male | 43.6% | 37.6% | 52.5% | 55.2% | 39.8% |
| Female | 56.4% | 62.4% | 47.5% | 44.8% | 60.2% |
| **Age** |  |  |  |  |  |
| 45 - 49 years | 11.9% | 16.3% | 4.7% | 3.4% | 11.7% |
| 50 - 54 years | 15.3% | 14.4% | 11.7% | 10.3% | 23.5% |
| 55 - 59 years | 14.0% | 19.8% | 9.3% | 17.2% | 8.7% |
| 60 - 64 years | 21.3% | 20.9% | 23.3% | 27.6% | 24.0% |
| 65 - 69 years | 20.7% | 14.4% | 27.6% | 24.1% | 21.9% |
| 70 - 74 years | 16.7% | 14.1% | 23.3% | 17.2% | 10.2% |
| **Geographic location** |  |  |  |  |  |
| Major Cities of Australia | 73.6% | 73.0% | 73.9% | 75.9% | 72.4% |
| Inner Regional Australia | 19.3% | 17.1% | 21.0% | 20.7% | 21.4% |
| Outer Regional Australia | 6.7% | 9.5% | 4.3% | 3.4% | 5.1% |
| Remote Australia | 0.3% | 0.4% | 0.4% | 0.0% | 0.5% |
| Very Remote Australia | 0.2% | 0.0% | 0.4% | 0.0% | 0.5% |
| **Ethnicity** |  |  |  |  |  |
| White/Caucasian | 92.5% | 90.1% | 94.6% | 93.1% | 93.9% |
| Asian | 3.3% | 4.9% | 1.9% | 3.4% | 2.0% |
| Middle Eastern | 1.1% | 1.5% | 1.2% | 0.0% | 1.0% |
| Chinese | 0.3% | 0.0% | 0.4% | 3.4% | 0.5% |
| Other | 2.7% | 3.4% | 1.9% | 0.0% | 2.6% |
| **Country of birth** |  |  |  |  |  |
| Australia | 75.3% | 71.9% | 79.0% | 72.4% | 80.6% |
| Other | 23.7% | 26.6% | 20.2% | 27.6% | 19.4% |
| Rather not say | 1.0% | 1.5% | 0.8% | 0.0% | 0.0% |
| **Languages spoken at home other than English** |  |  |  |  |  |
| No, English only | 94.3% | 91.6% | 96.9% | 96.6% | 94.9% |
| Yes, Other | 5.7% | 8.4% | 3.1% | 3.4% | 5.1% |
| **Marital status** |  |  |  |  |  |
| Never married | 13.7% | 13.3% | 12.1% | 13.8% | 18.9% |
| Married/Living with partner | 63.2% | 66.9% | 61.9% | 69.0% | 52.0% |
| Widowed/divorced/separated | 23.1% | 19.8% | 26.1% | 17.2% | 29.1% |
| **Smoking status** |  |  |  |  |  |
| No, never | 45.7% | 48.7% | 45.1% | 31.0% | 41.8% |
| Yes, quit more than a year ago | 35.4% | 32.3% | 37.0% | 58.6% | 34.2% |
| Yes, quit in last 12 months | 1.4% | 1.1% | 2.3% | 0.0% | 2.0% |
| Yes (social) | 4.0% | 5.7% | 2.3% | 0.0% | 2.6% |
| Yes (current) | 13.5% | 12.2% | 13.2% | 10.3% | 19.4% |
| **Body mass index (BMI)** |  |  |  |  |  |
| Mean BMI (kg/m^2^) | 29.5 | 27.7 | 31.3 | 28.4 | 31.1 |
| Underweight (below 18.5) | 1.3% | 1.9% | 0.8% | 0.0% | 0.5% |
| Normal (18.5 - 24.9) | 23.9% | 32.7% | 14.8% | 20.7% | 18.4% |
| Overweight (25 - 29.9) | 34.4% | 37.3% | 32.3% | 44.8% | 26.0% |
| Obese (30 and above) | 40.4% | 28.1% | 52.1% | 34.5% | 55.1% |
| **Education** |  |  |  |  |  |
| Less than Year 12 | 18.9% | 14.4% | 23.0% | 24.1% | 25.5% |
| Senior Secondary School Certificate of Education | 18.3% | 18.6% | 18.7% | 10.3% | 15.8% |
| Vocational qualification | 33.6% | 36.9% | 30.0% | 24.1% | 33.7% |
| Bachelor degree | 18.0% | 17.1% | 17.9% | 20.7% | 16.3% |
| Postgraduate qualification | 10.2% | 12.5% | 8.9% | 20.7% | 7.1% |
| Rather not say | 1.0% | 0.4% | 1.6% | 0.0% | 1.5% |
| **Household income (prior year, pre-tax)** |  |  |  |  |  |
| Under $10,000 | 1.4% | 0.4% | 1.6% | 6.9% | 2.6% |
| $10,000-$49,999 | 34.7% | 28.1% | 38.1% | 41.4% | 44.9% |
| $50,000-$74,999 | 19.9% | 17.5% | 23.3% | 13.8% | 19.4% |
| $75,000-$99,999 | 12.1% | 13.7% | 10.1% | 17.2% | 10.7% |
| $100,000-$149,999 | 14.0% | 17.9% | 10.9% | 10.3% | 9.7% |
| $150,000 or more | 7.3% | 11.0% | 4.7% | 3.4% | 4.6% |
| Rather not say | 10.5% | 11.4% | 11.3% | 6.9% | 8.2% |
| **Employment status** |  |  |  |  |  |
| Full-time employed | 21.8% | 31.9% | 15.2% | 20.7% | 14.8% |
| Part-time employed | 16.1% | 19.0% | 12.8% | 6.9% | 14.3% |
| Unpaid work (e.g. volunteering) | 2.7% | 1.1% | 2.7% | 10.3% | 5.6% |
| Caregiver (e.g., children, elderly) | 4.1% | 3.8% | 2.3% | 3.4% | 6.6% |
| Full-time student | 0.6% | 0.4% | 0.4% | 0.0% | 1.0% |
| Part-time student | 0.2% | 0.0% | 0.4% | 0.0% | 0.0% |
| Retired | 44.1% | 33.5% | 58.0% | 51.7% | 42.9% |
| Other | 10.4% | 10.3% | 8.2% | 6.9% | 14.8% |
| **Medical insurance** |  |  |  |  |  |
| No | 42.2% | 36.5% | 41.2% | 44.8% | 56.6% |
| Yes | 57.8% | 63.5% | 58.8% | 55.2% | 43.4% |

* Due to the small sample sizes (N<30), statistical analysis has not been undertaken within this population.
CV, cardiovascular, GI, gastrointestinal, OA, osteoarthritis.
Statistically significant (p<0.05) differences between the proportions of participants within any given metric are noted in the tables, with blue indicating a significantly higher proportion and red indicating a significantly lower proportion versus the complement.
